# The 2010 expansion of activity-based hospital payment in Israel: an evaluation of effects at the ward level

**DOI:** 10.1186/s12913-019-4083-4

**Published:** 2019-05-08

**Authors:** Ruth Waitzberg, Wilm Quentin, Elad Daniels, Vadim Perman, Shuli Brammli-Greenberg, Reinhard Busse, Dan Greenberg

**Affiliations:** 1 The Smokler Center for Health Policy Research, Myers-JDC-Brookdale Institute, JDC Hill, P.O.B. 3886, 91037 Jerusalem, Israel; 2 Department of Health Systems Management, School of Public Health, Faculty of Health Sciences, Ben-Gurion University of the Negev, Beer-Sheva, Israel; 3 Department of Health Care Management, Faculty of Economics & Management, Technical University Berlin, Berlin, Germany; 4 European Observatory on Health Systems and Policies, Brussels, Belgium; 5 Planning, Budgeting and Pricing division, Ministry of Health, Jerusalem, Israel; 6 School of Public Health, University of Haifa, Haifa, Israel

**Keywords:** Activity-based payments, Procedure-related group (PRG), Diagnosis-related group (DRG), Hospital financial incentives, Health-policy reform, Provider-payment reform

## Abstract

**Background:**

In 2010, Israel intensified its adoption of Procedure-Related Group (PRG) based hospital payments, a local version of DRG (Diagnosis-related group). PRGs were created for certain procedures by clinical fields such as urology, orthopedics, and ophthalmology. Non-procedural hospitalizations and other specific procedures continued to be paid for as per-diems (PD). Whether this payment reform affected inpatient activities, measured by the number of discharges and average length of stay (ALoS), is unclear.

**Methods:**

We analyzed inpatient data provided by the Ministry of Health from all 29 public hospitals in Israel. Our observations were hospital wards for the years 2008–2015, as proxies to clinical fields. We investigated the impact of this reform at the ward level using difference-in-differences analyses among procedural wards. Those for which PRG codes were created were treatment wards, other procedural wards served as controls. We further refined the analysis of effects on each ward separately.

**Results:**

Discharges increased more in the wards that were part of the control group than in the treatment wards as a group. However, a refined analysis of each treated ward separately reveals that discharges increased in some, but decreased in other wards. ALoS decreased more in treatment wards. Difference-in-differences results could not suggest causality between the PRG payment reform and changes in inpatient activity.

**Conclusions:**

Factors that may have hampered the effects of the reform are inadequate pricing of procedures, conflicting incentives created by other co-existing hospital-payment components, such as caps and retrospective subsidies, and the lack of resources to increase productivity. Payment reforms for health providers such as hospitals need to take into consideration the entire provider market, available resources, other – potentially conflicting – payment components, and the various parties involved and their interests.

## Highlights

- Israel intensified adoption of PRG payments to hospitals in 2010.
- Discharges increased in some, but decreased in other treatment-group wards.
- ALoS decreased more in treatment-group wards.
- Difference-in-difference results could not suggest causality of the reform for these changes.
- Payment reforms should consider the entire provider market and payment mechanism.

## Background

Payments to healthcare providers entail a set of economic incentives that influence provider behavior and decision-making [1, 2]. Israel adopted activity-based payments to replace per diems (PDs) and created codes for 30 common procedures as early as the 1990s [3]. The main objective of the change was to shorten waiting times for expensive procedures involving brief hospital stays, for which the PD payment was insufficient so that hospitals were discouraged from performing them [4]. Due to data and policy constraints, Israel chose procedure-related groups (PRGs) rather than Diagnosis Related Groups (DRGs) as the basis for measuring activity. PRGs differ from DRGs in that they are defined based on type of treatment (surgical procedure) rather than diagnosis, and they are not adjusted for case-mix or disease severity [5].

In the past two decades, many OECD countries have shifted to hospital payments based on activity and adopted diagnosis-related groups (DRGs) as payment units but, unlike the Israeli case, their main objectives were to increase efficiency and transparency [6]. DRGs are still being adopted by mid-income countries [7]. In 2002, continuing the move towards activity-based payments, the Israeli Ministry of Health (MoH) created PRG codes for more procedures, in the same timing DRGs were introduced in some OECD countries such as Estonia, Germany and the Netherlands [6]. Since 2010, the MoH has further expanded the application of PRGs to several clinical specialties, in three main waves:

1. Wave 1: 2010–2012, trauma in orthopedics
2. Wave 2: 2013–2014, urology, general surgery, ophthalmology, head and neck surgery
3. Wave 3: 2015, orthopedics and MRI

The objectives of the 2010–2015 “PRG reform” mainly concerned transparency and a fair distribution of funds. The specific objectives were to refine the unit of payment and establish consistent costing and pricing mechanisms in order to reduce cost-price gaps, improve MoH ability to set policy and priorities, influence the supply of hospital services by adjusting prices, and conduct supervision and control [5]. Furthermore, PRG payments were expected to change the incentives for hospitals. If PD payments create incentives for longer stays, PRGs create incentives to perform more procedures and shorten the length of stay (LoS), to minimize operating costs and maximize profits.

Many studies have evaluated the impact of DRG-based payments in high- and middle-income countries on volume of activity, LoS, and quality of care [8–10]. In Israel, Shmueli and colleagues [11] examined the effects of the early introduction of PRG payments for five major procedures, one year after implementation in 1990. They found that the volume of activity increased for two procedures, remained unchanged for two others, and decreased for the last one. Regarding LoS, there was a modest decrease in three procedures and a significant decrease in the other two. A later study evaluated the effect of incorporating the time interval between hospitalization and treatment (of hip fractures) in the PRG tariff (maximum fees are paid for patients operated within 48 h, for those operated later, payments are significantly lower); it found that the LoS decreased following this change in payment method [12].

Since then, no study has evaluated the effects of the later adoption of PRGs on hospital activity. The effects of the 2010-reform thus remain largely unknown, preventing evidence-informed discussion of its benefits and challenges. The current study adds to the previous literature both by analyzing the changes that have occurred since then, and extending it, by examining all the hospital data and including all the activities performed at the ward level.

### Background on the Israeli case and the hospital market

Since 1995, Israel has had a national health insurance (NHI): four competing, non-profit health plans (HPs) are responsible for providing and managing a broad benefits package determined by the government. The HPs provide care in the community and purchase hospital services for their members.

**Fig. 1:**
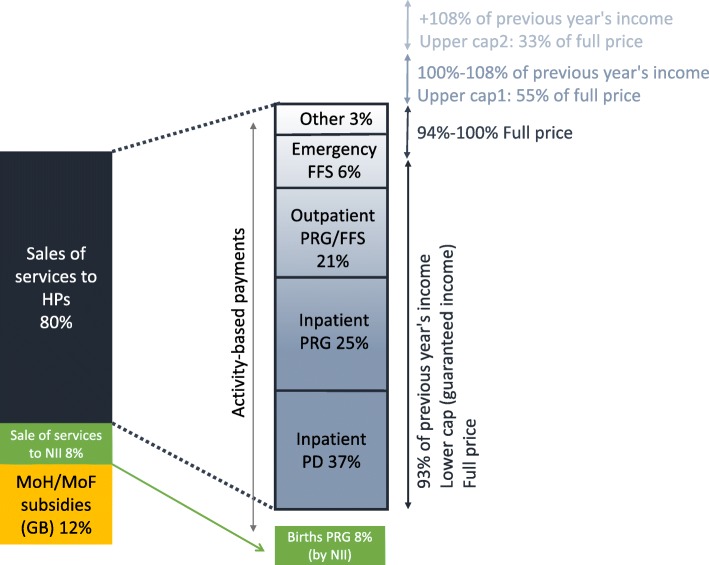
Public hospitals' sources of income, types of payment and cap mechanism Notes: H: hospitals, MoH: Ministry of Health, MoF: Ministry of Finance, HP: health plan, NII: National Insurance Institute, GB: global budgets, PD: per diem, PRG: procedure-related group, FFS: fee-for-service.

Of the 44 general hospitals in Israel, 35 are non-profit and owned by the Ministry of Health (MoH), the municipalities, the Clalit HP or NGOs. These are considered “public hospitals.” The other nine are smaller, for-profit hospitals, and operate 3% of the beds. The main source of income of Israeli public hospitals is the sale of services to HPs and the National Insurance Institute (NII) (see left-hand column in Fig. 1). Hospital reimbursement rates are determined by a joint MoH and Ministry of Finance (MoF) pricing committee, stipulated in the “Price List for Ambulatory and Inpatient Services.” This maximum list-price (tariff) also determines the type of payment, which can be PD; per activity (PRG); or fee-for-service (FFS) (see right-hand column in Fig. 1). There are currently 24 PD rates according to ward type and length of stay (the tariff of the first three days is higher than the tariff of the subsequent days), about 320 PRG codes, and more than 1600 ambulatory service codes. In 2015, 25% of the gross revenue of hospitals was for inpatient care paid as PRGs, 37% for inpatient care paid as PDs, 21% for ambulatory care paid as FFS or PRGs, 8% for births paid as PRGs, 6% for emergency care paid as FFS, and 3% from other sources such as the Ministry of Defense or the military [13].

The sale of services covers hospital marginal costs and some fixed costs such as physician salaries. Public hospitals also receive “prospective subsidies” in the form of global budgets from the MoH to cover part of the other fixed costs such as infrastructure and equipment. Furthermore, the government provides “retrospective subsidies” for public hospitals with a financial deficit at the end of each year. Both subsidies are negotiated with both the MoH and MoF. Overall, hospitals received about NIS 1500 million, which roughly represents 12% of their income from government subsidies (yellow box in Fig. 1) [13].

Israeli public hospitals are subject to two major income constraints. The first, put in place in 2005, is a cap mechanism; the MoH sets annual caps on hospital revenues from each HP to each hospital (see vertical arrows at right-hand side of Fig. 1). In recent years, caps have been set as a floor (lower cap bound) and a ceiling (upper cap bound), and are updated every three years. The floor is a minimum payment amount, set in 2016 as 93% of the previous year’s expenditure for each HP to each hospital. If an HP consumes services that, at “list prices”, would have an aggregate cost of less than the lower cap, the HP pays 93% of the previous year’s expenditure to the hospital in any case. The ceiling is a maximum payment amount and when an HP spends more than this threshold, it pays only a percentage (less than 100%) of the full price [14]. Towards the end of the financial year, once the upper bound of the cap is reached, there is an incentive for HP to refer patients, when possible (e.g., for elective procedures) to the hospital as they do not pay the full price for these services. In 2016, the hospitals’ net income was 15% lower than the potential gross income due to discounts related to the cap mechanism [13]. The second constraint is a negotiated alternative reimbursement contract between an HP and a hospital that may supplant the official cap, with such contracts entailing discounts that vary across HPs and hospitals [4]. In 2015, individual discounts represented 4% of the hospitals’ gross income [13].

Acute hospital care in Israel has a high rate of overcrowding, one of the highest among OECD countries. Compared with the OECD average, Israeli hospitals function with half the rates of acute-care beds and nurses per population. In 2017, the average length of stay (ALoS) in Israeli hospitals was 4 days, one of the shortest, and occupancy rates of acute-care beds was one of the highest among OECD countries, reaching almost full capacity, 93%. Nonetheless, the number of discharges per 100,000 population in Israel is almost the same as the OECD average [15, 16].

### Objectives

Our objective was to examine changes in the volume of activity, measured by the number of discharges and ALoS, in hospitals following the PRG reform. The focus was on changes on the macro/system level, aiming at draw generalizable conclusions about the payment-policy change rather than an examination of the impact on specific procedures or hospitals. Since PRG codes were created in waves by clinical area, we hypothesized increasing volumes and decreasing ALoS in the clinical areas for which PRG codes were created. Our analysis focuses on hospital wards as a proxy for such clinical areas.

Economic theory suggests that hospitals react to economic incentives derived from payment mechanisms [17]. Peleg and colleagues [12] show that immediately after the adoption of the refined PRG codes, Israeli hospitals reacted by costing the wait between injury and surgery for timing of hip fracture procedures. Based on international experience [9], it is quite plausible that this was a reaction to the PRG reform. However, in contrast to other OECD countries, where DRGs were adopted to improve efficiency, Israeli hospitals operated with relatively limited resources even before the adoption of PRGs in 2010, potentially limiting the hospitals’ capacity for increased activity. The analysis of the effect of the PRG reform on hospital volume and ALoS, in so different an environment, thus provides an interesting case.

## Methods

The analysis is based on data on inpatient care provided by the MoH for all public general hospitals. Our observations were of hospital wards for the years 2008–15, two years prior to the first wave of reform (2008–10) and two years after each wave (2011–13, period1; 2014–15, period2). The data were aggregate; they did not apply to individual patients or the level of procedures. Derived from HP-hospital accounts reported to the MoH, the data included the following variables: ward type, hospital code, dummy for hospital location in the periphery, ownership (government, HP or NGO), number of annual discharges per ward, and annual ALoS per ward. The data related to procedural acute-care wards. They excluded medical wards (such as internal medicine, neurology and pediatrics) since in medical wards there are few procedures and PRG payments. We further excluded long-term care wards such as psychiatry, rehabilitation and geriatrics; intensive-care and observation wards since they are not good proxies for specific medical areas; and obstetric wards because deliveries are paid for by the NII, rather than HPs and the reimbursement mechanism described above does not apply (there are no caps, subsidies or negotiation of discounts with hospitals).

To investigate changes in the number of discharges and in ALoS, we chose a difference-in-differences (DiD) approach that compares treatment and control groups for the period before and after waves 1 and 2 of the PRG reform. Although there were PRG codes before 2010, most of them were created in the 1990s and in 2002. Thus, their impact occurred before 2008 and they should not blur the effects of the 2010–14 waves. The treatment group was composed of procedural wards for which blocks of PRG codes were created between 2010 and 2014. The control group was composed of procedural wards for which no PRG codes were created in the same period. We analyzed the effects of the PRG reform on the number of discharges and the ALoS for waves 1 and 2 separately (Eqs. 1 and 2 below), and then the effects on each ward (Eqs. 3 and 4), since the reform might have affected each ward in a different manner, direction or intensity. The first wave refers to codes created in 2010–12 for orthopedic procedures; the second wave, for codes created from July 2013 to January 2014 for procedures in general surgery, urology, ophthalmology, and head and neck surgery. The 2015 wave is not analyzed in this work as not enough time has passed to observe its effects. The control group consists of pediatric surgery, cardiovascular surgery, vascular surgery, plastic surgery, gynecology, neurosurgery, oral and maxillofacial surgery wards.

The data were analyzed using SPSS 24 version (SPSS-IBM). We calculated the number of annual discharges and the ALoS for each treatment and control group. The ALoS was weighted for size of ward (measured by the number of discharges). The weighting was performed to balance the relative influence of each ward on the ALoS. For example, the relative importance of a small ward with a longer ALoS is smaller than that of a large ward with a shorter ALoS. The changing trends in the volume of discharges and in ALoS are depicted in graph form.

To verify the independent impact of each wave of PRG reform on the dependent variables (the number of annual discharges and the ALoS per ward), we performed a DiD analysis. We conducted the analysis using ordinary least squares (OLS) regressions. To mitigate skewness of the dependent variables, we transformed them with a natural logarithm. We controlled for hospital, wards and year fixed effects.

We clustered the data by wards, given that the same type of ward in different hospitals should exhibit more robust homogeneity than different wards within each hospital. We built one regression for each dependent variable following the models below:

1 $$ {lndis}_{it}=\alpha +{\beta}_1 wave{1}_{it}+{\beta}_2 wave{2}_{it}+{\gamma}_1 period{1}_{it}+{\gamma}_2 period{2}_{it}+{\delta}_1{\left( wave1\ast period1\right)}_{it}+{\delta}_2{\left( wave1\ast period2\right)}_{it}+{\delta}_3{\left( wave2\ast period2\right)}_{it}+{C}_i+{t}_i+e $$

2 $$ {lnALoS}_{it}=\alpha +{\beta}_1 wave{1}_{it}+{\beta}_2 wave{2}_{it}+{\gamma}_1 period{1}_{it}+{\gamma}_2 period{2}_{it}+{\delta}_1{\left( wave1\ast period1\right)}_{it}+{\delta}_2{\left( wave1\ast period2\right)}_{it}+{\delta}_3{\left( wave2\ast period2\right)}_{it}+{C}_i+{t}_i+e $$

For both equations, α represents the intercept that captures the model’s unexplained variance. The *wave* variable is a dummy for the two sets of treatment and control groups (wave 1 for orthopedics vs. the others; and wave 2 for general surgery, urology, ophthalmology, head and neck surgery vs. other procedural, non-participant wards). We examined the short- and long-term impact of wave 1 (orthopedics) and the short-term impact of wave 2 represented in the equation by *period 1*, which refers to 2011–13; and *period* 2, which refers to 2014 and 2015*.* The coefficient of interest is δ, the DiD estimator, as it captures the treatment groups of wards in the period after each reform wave: δ(wave*period). *δ*_1_ and *δ*_2_ capture the short- and long-term effects of the first wave, respectively. *δ*_3_ captures the short-term effects of the second wave of PRG expansion. The control variables, C_i,_ are the fixed effects for hospitals and wards, and T_i_, for time trends (year).

In a more refined analysis, we examined the reform’s effects in each ward separately, according to the following models:

3 $$ {Indis}_{it}=\alpha +{\beta}_1{orthopedics}_{it}+{\beta}_2{gensurg}_{it}+{\beta}_2{urology}_{it}+{\beta}_2{ophtalmology}_{it}+{\beta}_2{headneck}_{it}+{\gamma}_1 period{1}_{it}+{\gamma}_2 period{2}_{it}+{\delta}_1{\left( orthopedics\ast period1\right)}_{it}+{\delta}_2{\left( orthopedics\ast period2\right)}_{it}+{\delta}_3{\left( gensurg\ast period2\right)}_{it}+{\delta}_4{\left( urology\ast period2\right)}_{it}+{\delta}_4{\left( ophthalmogy\ast period2\right)}_{it}+{\delta}_6{\left( headneck\ast period2\right)}_{it}+{C}_i+{t}_i+e $$

4 $$ {lnALoS}_{it}=\alpha +{\beta}_1{orthopedics}_{it}+{\beta}_2{gensurg}_{it}+{\beta}_2{urology}_{it}+{\beta}_2{ophtalmology}_{it}+{\beta}_2{headneck}_{it}+{\gamma}_1 period{1}_{it}+{\gamma}_2 period{2}_{it}+{\delta}_1{\left( orthopedics\ast period1\right)}_{it}+{\delta}_2{\left( orthopedics\ast period2\right)}_{it}+{\delta}_3{\left( gensurg\ast period2\right)}_{it}+{\delta}_4{\left( urology\ast period2\right)}_{it}+{\delta}_4{\left( ophthalmogy\ast period2\right)}_{it}+{\delta}_6{\left( headneck\ast period2\right)}_{it}+{C}_i+{t}_i+e $$

## Results

**Fig. 2:**
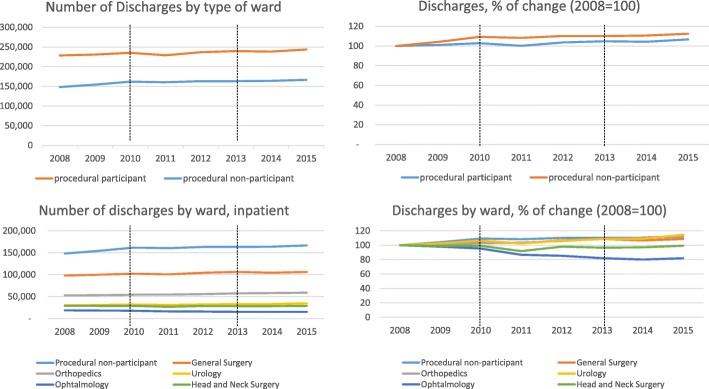
Number of discharges, by type of wards

**Fig. 3:**
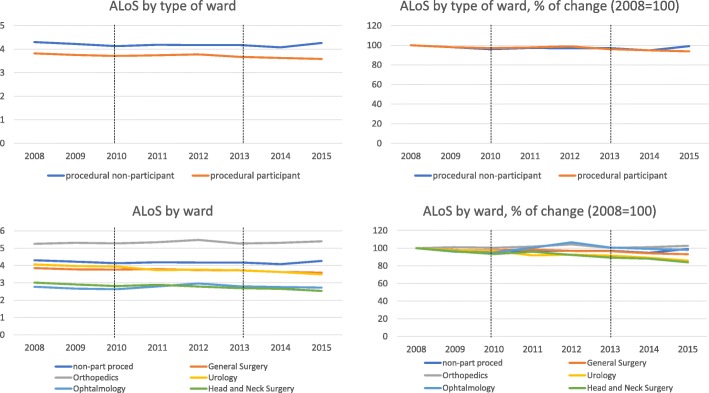
ALoS, by type of wards

Table 1 summarizes the changes in the number of discharges and ALoS in the study period, by treatment and control group. Figures 2 and 3 also show trends over time. Since we excluded some wards from the analysis, the number of discharges is smaller than the national data reported by the MoH, ranging from 376,480 in 2008 to 410,160 in 2015, an increase of 9%; the ALoS remained constant at 4.1 days. Our findings show that the trends and changes in ALoS and the number of discharges over time, in our dataset, are the same as that recorded by the MoH.

**Table 1:** Summary of changes in number of discharges and ALoS, 2008–2015, by ward

		Procedural non-participant	Procedural participant	General surgery	Orthopedics	Urology	Ophthalmology	Head and neck surgery
Discharges	2008	148,077	228,403	97,822	52,711	30,190	18,590	29,090
	2015	166,478	243,681	106,219	58,799	34,563	15,219	28,881
	change	18,401	15,278	8397	6088	4373	− 3371	− 209
	% change	12%	7%	9%	12%	14%	−18%	−1%
ALoS	2008	4.30	3.82	3.85	5.25	4.06	2.78	3.02
	2015	4.26	3.59	3.59	5.39	3.49	2.72	2.53
	change	−0.04	−0.24	−0.26	0.14	−0.57	−0.06	−0.48
	% change	−1%	−6%	−7%	3%	−14%	−2%	−16%

Notes: *ALoS* = average length of stay, ALoS are weighted by ward size. Reform participant (treatment) wards consist of general surgery, urology, ophthalmology, head and neck surgery. Non-participant (control group) include pediatric surgery, cardiovascular surgery, vascular surgery, gynecology, neurosurgery, oral and maxillofacial surgery wards

When analyzing the changes in the number of discharges by treatment and control group, we see that it increased more markedly in control (non-participant) wards (12%) compared with treatment (participant) wards (7%). However, while refining the analysis to focus on specific treatment wards, we observed an increase in volume in the general surgery, orthopedics and urology wards, but a sharp decrease in ophthalmology, and no change in the head and neck surgery ward, despite the high rate of population growth of 1.8% annually. The ALoS decreased more sharply in participant wards (6%) than in non-participant wards (1%). These results are in line with our hypothesis that the adoption of PRGs increases volume and shortens length of stay. A more in-depth focus on each participant ward shows that the ALoS decreased sharply in urology and in head and neck surgery (14 and 16% respectively), but remained almost unchanged in orthopedics and urology.

### Multivariable DiD analysis

The results of the multivariable DiD analysis are presented in Table 2. Our coefficients of interest, δ, are the DiD estimates of the interaction between the treatment dummies (waves, coded with received treatment = 1) and our period variables (2011–13 and 2014–15). The table shows results for model 1 (comparing treatment and control wards) and model 2 (analysis at the ward level) for both the natural logarithm of number of discharges and the ALoS. In all models, the DiD estimates were small and not significant with the exception of the head and neck surgery ward where the discharges and ALoS decreased by 24% and 9%, respectively.

**Table 2:** Results of DiD analysis

DiD coefficient (δ)	lndis						lnALoS					
	Model 1			Model 2			Model 1			Model 2		
	Estimate δ	(CI)		Estimate δ	(CI)		Estimate δ	(CI)		Estimate δ	(CI)	
Wave1*period1	0.068	(− 0.011	0.147)	0.068	(− 0.011	0.147)	0.017	(− 0.014	0.048)	0.017	(− 0.014	0.048)
Wave1*period2	0.026	(− 0.141	0.193)	0.026	(− 0.141	0.193)	−0.001	(− 0.087	0.084)	− 0.001	(− 0.087	0.084)
Wave2*period2	−0.097	(− 0.291	0.097)				− 0.055	(− 0.141	0.030)			
General surgery*period2				−0.030	(−0.194	0.134)				−0.043	(− 0.126	0.039)
urology*period2				0.030	(−0.103	0.164)				−0.063	(−0.139	0.012)
Ophthalmology*period2				−0.151	(−0.307	0.005)				−0.025	(− 0.105	0.054)
head neck*period2				−0.239*	(−0.409	− 0.070)				−0.086*	(− 0.168	−0.004)
R Square	0.48			0.59			0.47			0.51		
Number of cases	1828			1828			1828			1828		
number of hospitals	29			29			29			29		

Notes: *lndis* = natural logarithm of number of discharges, *lnALoS* = natural logarithm of average length of stay, *wave 1* = orthopedics, *wave 2* = general surgery, urology, \’/ ophthalmology, head and neck surgery; period 1 = 2011–2013; period 2 = 2014–2015; CI = 95% Confidence Interval in parenthesis; *p < 0.05; **p < 0.01. Regression OLS, clustered by ward. ALoS were weighted by ward size. Model 1 includes reform waves as predictors, model 2 includes each ward separately as predictors

## Discussion

In our study, despite the changes seen in the descriptive statistics, the DiD analysis could not demonstrate causality between the PRG reform and the changing volume of hospital activities or the ALoS, at least not when comparing inpatient activities at the ward level. It is likely that the adoption of PRGs created incentives to increase the volume of specific procedures or to change the quality of care. However, an examination of such an impact was beyond the scope of this study. Rapid population growth and aging may explain part of the volume increases while technological innovations that allow for shorter hospital stays may be related to the decrease in ALoS.

One plausible explanation for the counterintuitive finding of no significant PRG-reform effect, which deserves further analysis, is the difference between PRG payments and the previous PD payments for the same procedure. If the PRG tariff is lower than the original PD payment (calculated as the PD rate times the length of stay), the reform might not create a strong incentive to increase volume. This might be particularly true in Israel where the pricing mechanism is constrained and somewhat distorted due to a budget-neutral requirement that may lead to inaccurate prices [5]. In a 2018 qualitative study, hospital managers, ward directors, and surgeons reported that indeed, most PRG-paid procedures were underpriced [18].

A second possible explanation is that Israeli hospitals already worked under pressure before adopting PRGs, leaving little room for further increase of activities or reduction of length of stay. It is possible that hospitals simply do not have the necessary resources to treat more patients. As mentioned, the rates of hospital beds per population is one of the lowest among OECD countries. Rates of physicians have declined and are expected to drop below the OECD average in the coming decade [19, 20]. There is a particular shortage of anesthesiologists and surgeons, causing a bottleneck for various procedures, at least in the short-term [21].

Finally, a third explanation is that other components of the hospital payment system, such as caps and subsidies, modify the incentives created by PRG payments. Caps on hospital income can deter hospitals from increasing activities beyond the ceiling. Retrospective subsidies avert hospital collapse, but also reduce their fiscal responsibility, transferring the risk to the MoH. Subsidies may blur the effects of the PRG reform on hospital activities if they are not financially responsive. Feldhaus and Mathauer [22] also conclude that mixed or blended provider-payment mechanisms may restrain economic incentives. They stress that the effects of payment reforms are highly context-specific.

Our study adds to the literature on activity-based payment and its economic incentives. Diagnosis-related groups (DRGs) were originally developed in the US to incentivize hospitals to provide care more efficiently [23]. In the past two decades, many countries have shifted to hospital payment based on activity and adopted DRGs as a payment mechanism to improve efficiency while limiting incentives for patient selection [6]. In general, DRGs created economic incentives to cut costs and shorten ALoS. In Western European countries, DRGs also created incentives to maximize income by increasing the volume of (profitable) activities. Yet, there is evidence that DRGs also led to decreased volume (USA) or unchanged volume (Eastern European and Central Asian countries) [10]. Norton and colleagues [24] found, too, that overall, hospital ALoS did not decrease in the US when Medicaid introduced a flat episode payment for psychiatric patients in the 1990s, replacing PD. Our findings add another case of a country where the shift to activity-based payments did not seem to contribute to changing volume or a shorter ALoS.

Notwithstanding, the study has limitations that should be taken into account:

1. Analysis on the wards level may not be sufficiently refined to capture the effects of the reform. Currently, about a third of the activity in procedural wards is paid for by PRGs, which represent some 40% of the ward income. Possibly, an increase of patients with PRG-paid procedures in these wards was compensated for by a reduction in the number of patients treated with non-PRG-paid procedures.
2. Wards are not a perfect proxy for medical areas because about 15% of discharges are transfers between wards within a hospital, further diluting the potential effect of the PRG reform on a specific ward. Since the data were aggregated at the ward level, it was not possible to exclude the transfers from the dataset. Yet, the rate of transfers has remained constant over the study period, so the DiD analysis should overcome this limitation.
3. The study does not control for changes in population needs, preferences over time or the technological “menu” offered to patients due to ageing and changes in the case-mix. However, we believe that the DiD methodology overcomes the problem of ageing and case-mix changes as it affects all wards similarly.

## Conclusions

This is the first study to evaluate the impact of PRG reform in Israel on hospital activities, as measured by the number of inpatient discharges and the ALoS on the national level. The study did not find any significant effect of the PRG reform on ward-aggregate hospital inpatient volume or the ALoS. However, our inability to demonstrate a significant effect does not necessarily mean that the reform did not have any effect. As noted in the discussion, there are plausible explanations for the finding. These include conflicting incentives created by budget caps and subsidies, comparatively low PRG prices, and the limited capacity of hospitals to increase their volume because of limited resources.

Despite the counterintuitive evidence generated by this study, the possible absence of an effect for the reform is interesting, and warrants closer examination, because it has implications for both researchers and policymakers in Israel and in other countries. First, researchers conducting cross-country analyses should avoid simplistic assumptions about the effects of DRG-like payment components on volume and length of stay; the incentives of such payments are often modified by multiple, co-existing payment components of a given national hospital payment system. Second, policymakers engaging in hospital payment reforms need to take into consideration additional factors, such as the national hospital market, available resources, other – potentially conflicting – payment components, the various parties involved and their interests. More broadly speaking, unless payment reforms are accompanied by further measures that allow providers to respond to the changed incentives, e.g., by making available additional resources or allowing greater provider autonomy, the reforms are unlikely to lead to the intended changes.

## Abbreviations

ALoS: Average length of stay
DiD: Differences-in-differences
DRG: Diagnosis-related group
FFS: Fee for service
GB: Global budgets
HP: Health plan
LoS: Length of stay
MoF: Ministry of Finance
MoH: Ministry of Health
NGO: Non-governmental organization
NHI: National Health Insurance
NII: National Insurance Institute
NIS: New Israeli Shekels;
OECD: Organization for economic co-operation and development
PD: Per-diem
PRG: Procedure-related group
USA: United States of America
